# Contemporary Demographic Reconstruction Methods Are Robust to Genome Assembly Quality: A Case Study in Tasmanian Devils

**DOI:** 10.1093/molbev/msz191

**Published:** 2019-08-19

**Authors:** Austin H Patton, Mark J Margres, Amanda R Stahlke, Sarah Hendricks, Kevin Lewallen, Rodrigo K Hamede, Manuel Ruiz-Aravena, Oliver Ryder, Hamish I McCallum, Menna E Jones, Paul A Hohenlohe, Andrew Storfer

**Affiliations:** 1 School of Biological Sciences, Washington State University, Pullman, WA; 2 Department of Organismic and Evolutionary Biology, Harvard University, MA; 3 Institute for Bioinformatics and Evolutionary Studies, University of Idaho, Moscow, ID; 4 School of Natural Sciences, University of Tasmania, Hobart, Australia; 5 School of the Environment, Griffith University, Nathan, Australia; 6 Institute for Conservation Research, San Diego, CA

**Keywords:** whole-genome assembly, demographic history, simulation, SMC, SFS, Tasmanian devil

## Abstract

Reconstructing species’ demographic histories is a central focus of molecular ecology and evolution. Recently, an expanding suite of methods leveraging either the sequentially Markovian coalescent (SMC) or the site-frequency spectrum has been developed to reconstruct population size histories from genomic sequence data. However, few studies have investigated the robustness of these methods to genome assemblies of varying quality. In this study, we first present an improved genome assembly for the Tasmanian devil using the Chicago library method. Compared with the original reference genome, our new assembly reduces the number of scaffolds (from 35,975 to 10,010) and increases the scaffold N90 (from 0.101 to 2.164 Mb). Second, we assess the performance of four contemporary genomic methods for inferring population size history (PSMC, MSMC, SMC++, Stairway Plot), using the two devil genome assemblies as well as simulated, artificially fragmented genomes that approximate the hypothesized demographic history of Tasmanian devils. We demonstrate that each method is robust to assembly quality, producing similar estimates of Ne when simulated genomes were fragmented into up to 5,000 scaffolds. Overall, methods reliant on the SMC are most reliable between ∼300 generations before present (gbp) and 100 kgbp, whereas methods exclusively reliant on the site-frequency spectrum are most reliable between the present and 30 gbp. Our results suggest that when used in concert, genomic methods for reconstructing species’ effective population size histories 1) can be applied to nonmodel organisms without highly contiguous reference genomes, and 2) are capable of detecting independently documented effects of historical geological events.

## Introduction

Understanding historical population size trajectories has long been of interest among biologists. A variety of evolutionary processes can be inferred by studies of historical demography, including ancient dispersal (Li and Durbin 2011), speciation (Kliman et al. 2000), and population decline leading to present-day extinction risk (Caughley 1994). Recently, significant advances have been made in the development of population genomic methods to accomplish this goal. A suite of methods are now available that evaluate either whole-genome sequences (PSMC: Li and Durbin 2011; MSMC: Schiffels and Durbin 2014) or site-frequency spectra (SFS) (Stairway Plot: Liu and Fu 2015) obtained from genome-wide data sets. A key difference between these methods is to what extent explicit specification of demographic models is required. Methods reliant on the SFS are also agnostic to the physical distribution of sites and may be used with data for which sequences remain largely unassembled and at low density, such as with reduced representation approaches (e.g., ddRAD-seq; Peterson et al. 2012). In contrast, methods reliant on the sequentially Markovian coalescent (SMC) account for physical linkage thus necessitating assembled sequence data.

The first methods developed to use whole-genomes (PSMC and MSMC) require the a priori specification of demographic models in which population size is estimated in a semicontinuous or “stepped” trajectory through time. However, due to these models’ assumption of panmixia, the parameter being inferred is more accurately the inverse of the coalescence rate (Mazet et al. 2016). For consistency across methods however, we follow the convention of referring to these parameter estimates as the effective population size, *N*_e_. Similar to the PSMC and MSMC, the first methods developed to leverage the SFS (*δaδi* and Fastsimcoal2) produce stepped estimates of *N*_e_. These SFS methods require explicit specification of competing demographic models by discretizing time into a series of epochs, each characterized by a unique (unknown) value of *N*_e_ to be inferred. In turn, competing models of demographic expansion, contraction, or secondary contact can be tested.

Although many of the earliest applications of demographic reconstruction methods used the genomes of model organisms such as humans (Li and Durbin 2011; Kim et al. 2014), *Drosophila* (Duchen et al. 2013), and pigs (Groenen et al. 2012), application to nonmodel organisms soon followed (Barth et al. 2017; Mays et al. 2018; Westbury et al. 2018). With the increasing availability and affordability of high-throughput sequencing technology, whole-genome assemblies for nonmodel organisms are being published at an increasing rate (Ellegren 2014; Gouin et al. 2015; Matz 2017). Further, whole-genome data sets have recently become more commonly available for organisms of conservation concern (Fuentes-Pardo and Ruzzante 2017). Examples of their use include investigations of the decline of the Sumatran rhinoceros (Mays et al. 2018), the demographic history of yellow-fin tuna (Barth et al. 2017), and the low genomic diversity in the brown hyena (Westbury et al. 2018).

Nonetheless, genome assemblies of nonmodel organisms tend to be of lesser quality than those of their model-organism counterparts and are typically characterized by a high degree of fragmentation and limited annotation (Gouin et al. 2015). If use of these less contiguous genomes produce incorrect, or worse, biased inferences, downstream conservation measures may be misled. Although the impact of sequencing depth and filtering strategies on demographic reconstruction has been explored for PSMC (Nadachowska-Brzyska et al. 2016), an explicit test of the robustness of methods reliant on the SMC and SFS to reconstruct species’ demographic histories using genomes of varying assembly quality has yet to be performed.

Provided genome assemblies equivalent in length, but differing in the constituent number of scaffolds, we might expect methods dependent on the SFS and SMC to perform differently. Assuming the more fragmented assembly contains largely the same sites as in the less fragmented assembly, methods dependent on the SFS are expected to perform similarly across genomes, as they do not take into account linkage or contiguity among sites (Gutenkunst et al. 2009; Excoffier et al. 2013; Liu and Fu 2015). In contrast, methods dependent on the SMC, an approximation of the computationally intractable ancestral recombination graph (Wilton et al. 2015), walk along segments of the genome that are inferred to be contiguous, estimating a distribution of TMRCAs (time to most recent common ancestors). The distribution of TMRCAs is then used to produce piecewise-constant estimates of population size history (Li and Durbin 2011; Terhorst et al. 2017). Consequently, if contigs in a fragmented genome assembly are typically shorter than linkage disequilibrium blocks, SMC methods underestimate effective population sizes due to each segment possessing on an average less variation assuming a constant mutation rate, *μ*.

Each class of method performs best at different time scales. SMC methods tend to perform best at inferring relatively ancient demographic histories because the density of coalescent events tends to increase at deeper time scales (Terhorst et al. 2017). As a consequence of the limited number of recent coalescent events, SMC methods suffer from imprecise estimates toward the present (Liu and Fu 2015; Terhorst et al. 2017). In contrast, methods dependent on the SFS tend to perform best toward the present, as more ancient inferences are confounded by the impacts of recent demographic changes and saturation of sites present in the SFS (Liu and Fu 2015; Lapierre et al. 2017).

**Fig. 1.**
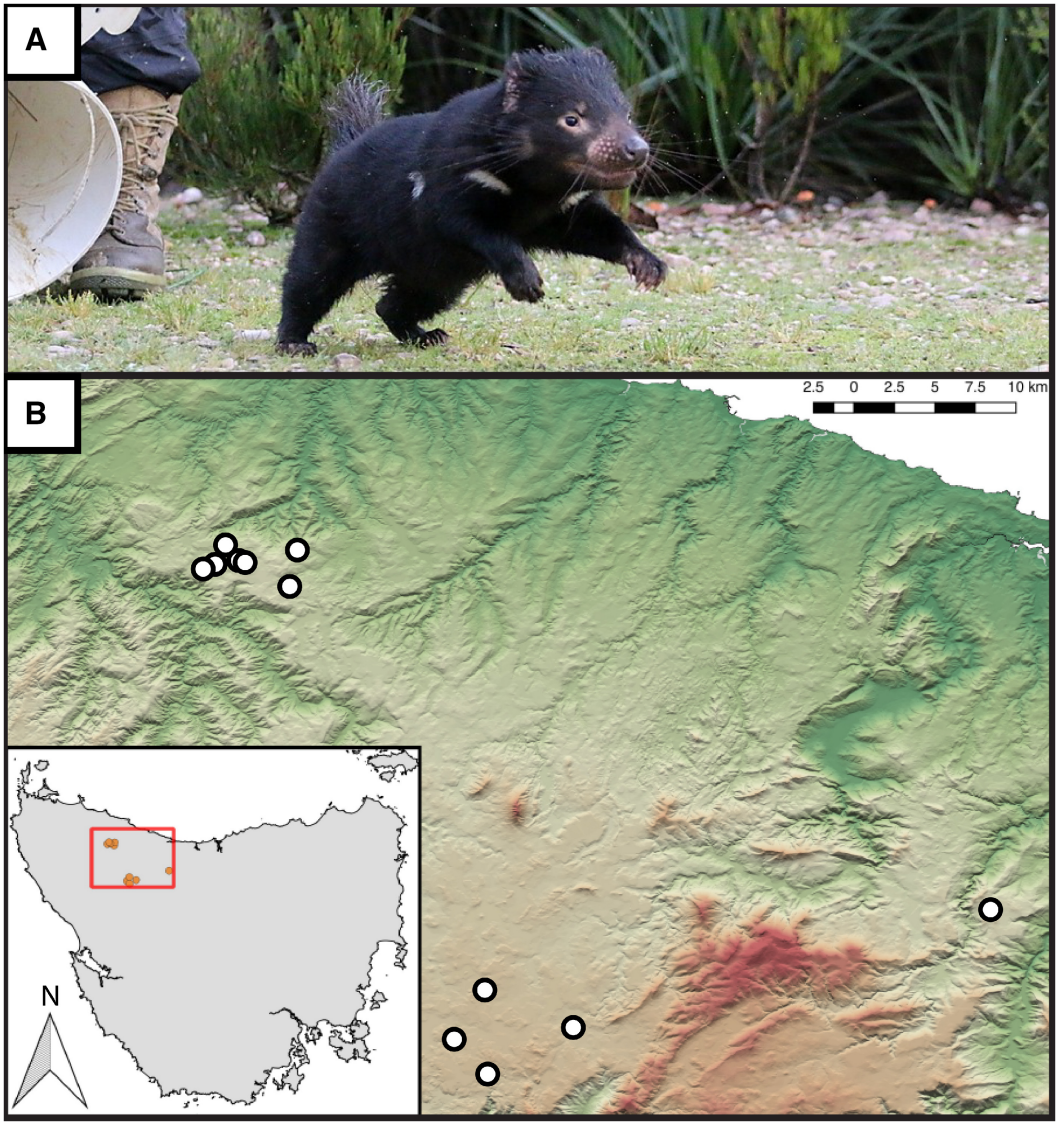
Sampling locations of Tasmanian devils sequenced for this study. (*A*) The Tasmanian devil. *Sarcophilus harrisii*. (*B*) The sampling locations for all 12 samples included in this study. (Photo credit: David Hamilton).

The Tasmanian devil, *Sarcophilus harrisii* (fig. 1), is ideally suited for a comparative study of how genome assembly quality affects estimates of effective population size because 1) a new reference genome is reported herein that reduced the extent of fragmentation relative to the original (Murchison et al. 2012), and 2) evidence of climatic, geological, and anthropogenic drives of historical demography are available for this species. Additionally, Tasmanian devils are of conservation concern (Hohenlohe et al. 2019), being threatened by Devil Facial Tumor Disease (DFTD) and DFT2 (Pearse and Swift 2006; Murchison 2008; Pye et al. 2016; Storfer et al. 2018), two of eight known transmissible cancers (Metzger and Goff 2016). Little is known about the impact of DFT2 (but see Caldwell et al. 2018; Stammnitz et al. 2018).

First identified in Northeastern Tasmania in 1996 (Hawkins et al. 2006), DFTD has since spread across >95% of the devils’ geographic range with localized devil population declines exceeding 80% (Lazenby et al. 2018). The impacts of DFTD have been so severe due to nearly universal susceptibility and case fatality rates approaching 100% (McCallum et al. 2009; Hamede et al. 2013, 2015). However, regression has been documented in a small number of devils (Wright et al. 2017; Margres, Ruiz-Aravena, et al. 2018), and recent genomic studies imply ongoing adaptation of the devil to this threat (Epstein et al. 2016; Caldwell et al. 2018; Margres, Jones, et al. 2018; Ruiz-Aravena et al. 2018). Understanding the demographic history of this imperilled species is thus a pressing matter.

The first reference genome for the Tasmanian devil (Murchison et al. 2012) was comprised of over 35,000 scaffolds with an L90 (number of scaffolds comprising 90% of the genome length) of 3,263, and an N90 (scaffold length at which the sum of all scaffolds this length or longer comprises 90% of the total genome) of 0.101 Mb (table 1; Murchison et al. 2012). Herein, we report a new reference genome which improves the L90 by nearly 10-fold and the N90 by over 20-fold (table 1). As the relationship between contig/scaffold size and linkage disequilibrium may impact demographic inference, and devils exhibit substantial LD (*R*^2^ ≈ 0.3 at 100 kb: see fig. S2 of Epstein et al. 2016), the improved assembly presented herein may enable more accurate estimates of effective population size.

**Table 1.** Summary of Tasmanian Devil Reference Genome Assemblies.

	Total Length (Mb)	L50 (Scaffolds)	L90 (Scaffolds)	N50 (Mb)	N90 (Mb)	Number of Scaffolds	Number of Scaffolds >1 kb	Longest Scaffold	Number of Gaps
2012-Assembly	3,174.71	520	3,263	1.847	0.101	35,975	35,966	5,315,331	3,490
2019-Assembly	3,177.42	129	422	7.751	2.164	10,010	10,001	28,347,192	30,589

Note.—2012-Assembly is the original reference genome assembly for *Sarcophilus harrisii* published by Murchison et al. (2012). 2019-Assembly is the assembly reported in this study produced by Dovetail Genomics leveraging the HiRise software platform and Chicago library method, using the 2012-Assembly as the input draft assembly. Note that, for every join, Dovetail genomics inserts a gap of 100 *N*’s. Thus, because 27,239 joins were made, the number of gaps increased correspondingly.

Mb, megabase pairs; bp, base pairs; L90, smallest number of scaffolds that comprises 90% of the total assembly length; N90, minimum scaffold length needed to cover 90% of the genome—sum of scaffolds this length or greater comprises 90% of the total length.

The evolutionary history of Tasmanian devils provides specific hypotheses regarding historical changes in effective population size. Historic geological and anthropogenic events have likely caused genetic bottlenecks in devils, perhaps an underlying factor in high susceptibility to DFTD (Miller et al. 2011). Tasmania has experienced two distinct pulses of connectivity to, and isolation from the Australian mainland (Lambeck and Chappell 2001). Initially connected to the mainland, the first period of isolation took place during the penultimate deglaciation (135–43 kybp) and persisted until the last glacial maximum (LGM: 25–14 kybp) when sea levels dropped, exposing the Bass Straight. Following the LGM, Tasmania has remained an isolated island through to the present day. Additionally, Tasmania has experienced two distinct rounds of human colonization, first by Aboriginal Australians (∼48.8 kybp: Tobler et al. 2017) and second by Europeans (215 ya: Owen and Pemberton 2005). Lastly, extreme El Niño-Southern Oscillation (ENSO) events occurred ∼3.15 kybp (Brown 2006). Consistent with these geologic and climatic events, ancient DNA and population genetic studies using MHC diversity (Morris et al. 2012), microsatellite markers (Brüniche-Olsen et al. 2014), and mtDNA (Miller et al. 2011) suggest devil population declines associated with these events (Miller et al. 2011; Morris et al. 2012; Brüniche-Olsen et al. 2014, 2018).

Using the documented historical events, we compared the performance of genome-scale methods for recovering associated changes in *N*_e_ using methods reliant on the SFS (SMC++, Stairway Plot) versus those leveraging the SMC (PSMC, MSMC, SMC++). By using the original reference genome of *S. harrisii*, as well as the new reference genome reported herein, we test the extent to which: 1) estimations of effective population sizes are robust to genome assembly quality within each method; 2) SFS and SMC methods differ in their ability to reconstruct ancestral effective population sizes; and 3) estimations of effective population sizes reflect historical geological, climatic, and anthropogenic events across the varying methods. To complement our empirical analyses, we explicitly test the robustness of each method to genome assembly fragmentation through simulation. Specifically, we replicate our empirical analyses using simulated whole genomes assuming a demography similar to the mean of the empirical estimates from the two devil reference genomes. We subsequently fragmented these simulated genomes to lesser, comparable, and more extreme levels than observed in our empirical assemblies.

## Results

### Improved Reference Genome Assembly

We produced an improved genome assembly for the Tasmanian devil using the Chicago library method (Dovetail Genomics, Santa Cruz, CA). Hereafter, we refer to the genome of Murchison et al. (2012) as the 2012-Assembly, and the genome presented herein as the 2019-Assembly. The Dovetail pipeline produced a vastly improved genome assembly according to several metrics (table 1). Whereas assembly sizes are approximately equal (2012-Assembly: 3,174.71 Mb, 2019-Assembly: 3,177.42 Mb) the number of scaffolds was reduced from 35,975 to 10,010, and the L90 was reduced from 3,263 to 422 scaffolds. The N90 improved from 0.101 Mb in the 2012-Assembly to 2.164 Mb in the 2019-Assembly (table 1). A summary of the assembly contiguities may be found in supplementary figure S1, Supplementary Material online. BUSCO completeness using the mammalia obd9 database for each assembly is shown in supplementary table S1, Supplementary Material online. This new reference genome has been made publicly available and has been deposited at RefSeq under BioProject PRJNA504904. We compare these two assemblies to other assemblies for both model and nonmodel organisms in table 2.

**Table 2.** Summary of Genome Assembly Quality/Contiguity for Both Model and Nonmodel Organisms, Including Those of Conservation Concern.

Organism	Assembly ID	Total Sequence Length (bp)	Number of Scaffolds	Scaffold N50 (Mb)	Scaffold L50	Number of Contigs	Contig N50 (Mb)
Koala	phaCin_unsw_v4.1	3,192.58	NA	NA	NA	1,907	11.5878
Common wombat	bare-nosed wombat	3,486.60	15,416	28.503	36	81,204	0.1131
Gray short-tailed opossum	MonDom5	3,598.44	5,223	59.810	18	72,674	0.1080
Tasmanian Devil (2012)	Devil_ref v7.0	3,174.69	35,974	1.847	520	237,291	0.0201
Tasmanian Devil (2019)	SarHar_Dovetail_2.0	3,177.40	10,010	7.751	129	238,513	0.0201
Dingo	ASM325472v1	2,439.83	2,444	34.446	22	3,121	5.3908
Horse	EquCab3.0	2,506.97	4,701	87.231	12	10,987	1.5028
Orca	Oorc_1.1	2,372.92	1,668	12.735	60	80,100	0.0703
Gorilla	gorGor4	3,063.36	40,730	81.227	15	170,105	0.0529
Chimpanzee	Clint_PTRv2	3,050.40	4,432	53.104	19	5,061	12.2686
Human	GRCh38.p12	3,099.71	472	67.795	16	998	57.8794
Polar bear	UrsMar_1.0	2,301.38	23,819	15.941	46	134,162	0.0465
Giant panda	AilMel_1.0	2,299.51	81,467	1.282	521	200,593	0.0399
Northern fur seal	ASM326570v1	2,706.87	14,230	31.507	23	52,955	0.1330
Southern white rhinoceros	CerSimSim1.0	2,464.37	3,087	26.278	30	57,824	0.0929

### Whole-Genome Resequencing

We resequenced the genomes of 12 devils trapped in northwestern Tasmania (fig. 1). The 12 resequenced genomes were subsequently aligned to the two alternative reference genomes. Across the 12 resequenced samples, using GATK, we identified 2,822,764 raw SNPs when aligning to the 2012-Assembly (Murchison et al. 2012) and 2,790,031 raw SNPs when aligning to the 2019-Assembly. However, following filtering with vcftools, we recovered 1,468,034 SNPs using the 2012-Assembly, as compared with 1,468,044 SNPs using the 2019-Assembly. The per-base mutation rate per-generation was estimated at 1.394808×10^–^^08^. For a description of how this estimate was obtained, see Materials and Methods (see Calculation of the Mutation Rate). Mean coverage across the 12 resequenced samples aligned to the 2012-Assembly was 26.27 ± 2.68. In contrast, mean coverage for the 2019-Assembly was 28.47 ± 2.90. A more detailed summary of per-sample read mapping and depth of coverage may be found in supplementary table S2, Supplementary Material online.

### Demographic Inference

#### Impact of Contiguity on Demographic Inference: Empirical Data

**Fig. 2.**
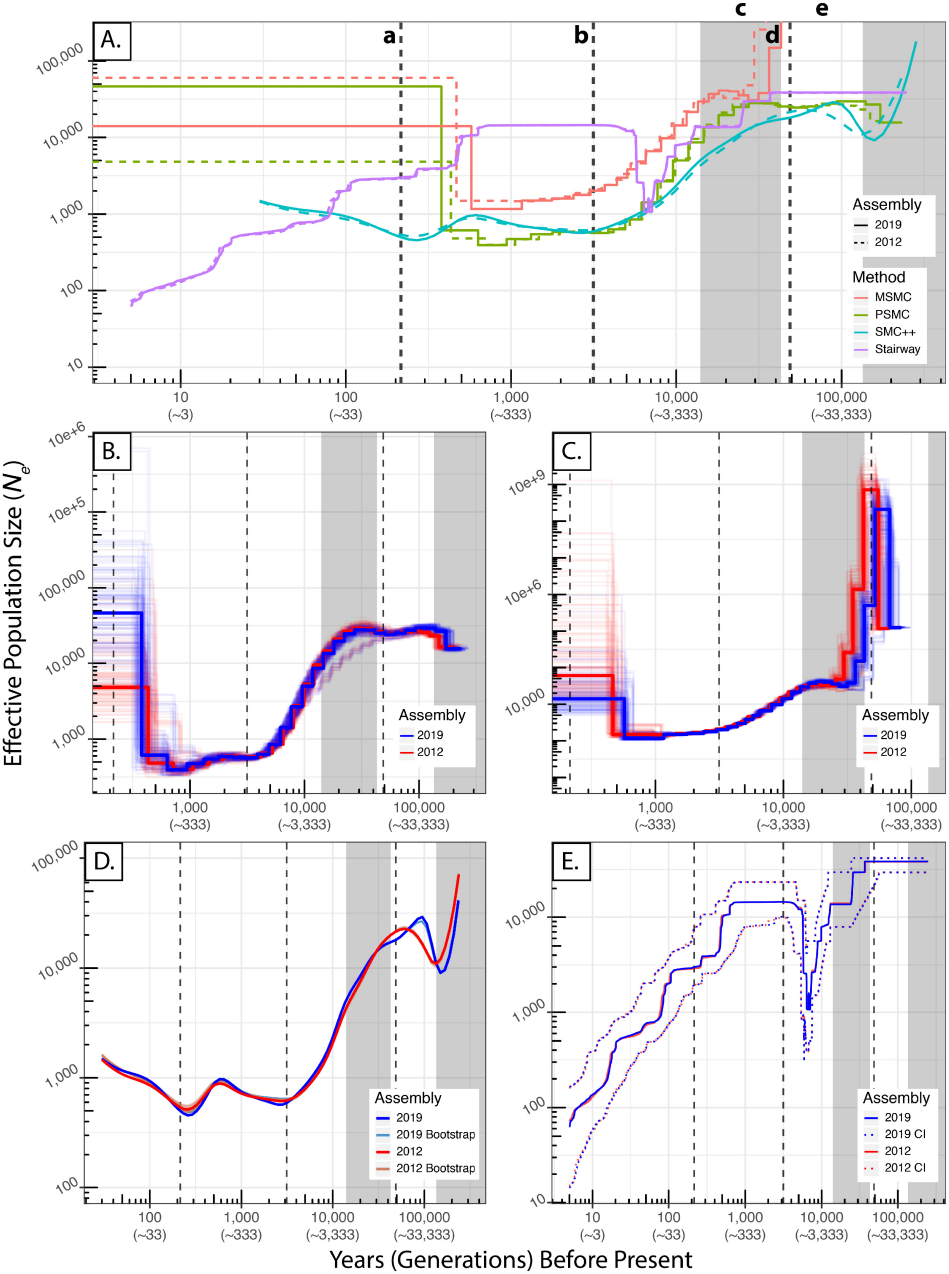
Estimates of the effective population size, *N*_e_, through time for Tasmanian devils using PSMC, MSMC, SMC++, and Stairway Plot. Panel (*A*) plots the estimates by all methods using each genome. Solid lines are the estimates of *N*_e_ using the 2019-Assembly, and dashed lines are the estimates obtained using the 2012-Assembly. Shaded bars indicate times during which Tasmania was connected to mainland Australia, whereas unshaded areas represent times in which Tasmania was isolated. Dotted vertical lines indicate other key climatic/anthropogenic events. Key events are as follows: a) colonization of Tasmania by the British (∼215 ya; Owen and Pemberton 2005), b) population bottleneck described by Brüniche-Olsen et al. (2014) (∼3.15 kybp), c) period of the last glacial cycle, including the Last Glacial Maximum (25 kybp) until the and separation of Tasmania from mainland Australia (14 kybp) (14 kybp; Lambeck and Chappell 2001), d) colonization of Tasmania by aboriginals (∼48.4 kybp; Lambeck and Chappell 2001; Tobler et al. 2017), and e) penultimate deglaciation (135–43 kybp) during which Tasmania was separated from mainland Australia (Lambeck and Chappell 2001). Panels (*B*–*E*) are the estimates with bootstrap replicates for PSMC, MSMC, SMC++, and Stairway Plot, respectively. Vertical dotted lines and shaded bars in these panels are interpreted the same as in panel (*A*). For PSMC and MSMC (panels *B* and *C*), bold lines are the median estimate for all 12 resequenced samples, whereas faint lines are individual bootstrap replicates; 10 replicates were conducted for each of the 12 samples. For SMC++ (panel *D*), the 25 bootstrap replicates are indicated using different colors to facilitate visualization due to the limited variation among replicates. For the Stairway Plot (panel *E*), solid lines correspond to median estimates using the site frequency spectrum of all 12 samples, whereas dotted lines correspond to the upper and lower bounds of the 95% CI. *X* axes differ for (*B*–*E*) to facilitate visualization of within-method and among-assembly comparison of results. Both years and number of generations (in parentheses) are reported on the *X* axis.

Estimates of historical *N*_e_ obtained using sequences aligned to each assembly were similar within each method (fig. 2*A* and supplementary fig. S2, Supplementary Material online). For PSMC (fig. 2*B*) and MSMC (fig. 2*C*), we report the median estimate (bold lines) for all 12 resequenced samples, and bootstrap replicates for individual samples (10 replicates per sample, shown as faint lines). Broadly speaking, the estimates obtained using all sequences aligned to one assembly fell within the confidence interval of the other during intermediate times (20,000–500 years before present: ybp, 6,666–166 generations before present: gbp). Note that the generation time of devils is 3 years (Pemberton 1990). PSMC results were similar among those obtained using resequenced samples aligned to each assembly from the most ancient estimates until ∼500 ybp (166 gbp), at which point estimates diverged dramatically (10-fold difference: fig. 2*B* and supplementary fig. S3, Supplementary Material online). MSMC follows a similar pattern, although the most recent (<∼500 ya, 166 gbp) estimates were also greatly divergent among assemblies (30-fold differences: fig. 2*C* and supplementary fig. S3, Supplementary Material online). Results from SMC++ were similar, with CIs of each estimate being extremely narrow (fig. 2*D* and supplementary fig. S2, Supplementary Material online). In contrast to the previous three methods, results obtained using the Stairway Plot were nearly identical between assemblies across the entire range of inference (fig. 2*E* and supplementary fig. S2, Supplementary Material online).

Bootstrapped estimates of *N*_e_ for each method revealed minimal differences in estimation uncertainty between genome assemblies for the Stairway Plot (fig. 2*E* and supplementary fig. S2, Supplementary Material online). Results for PSMC exhibited a similar pattern, however, there was greater variation albeit without a discernible trend (fig. 2*B* and supplementary fig. S2, Supplementary Material online). In contrast, the MSMC exhibited consistency only for intermediate time periods (20,000–500 ybp, 6,666–166 gbp) (fig. 2*C* and supplementary fig. S2, Supplementary Material online). Differences in the width of the 95% CI became dramatic at timescales >20,000 ybp (6,666 gbp) (supplementary fig. S2, Supplementary Material online), coincident with estimation of extremely large *N*_e_ (fig. 2). SMC++ was unique in that it exhibited a relatively consistent reduction in the width of the 2019-Assembly’s 95% CI through time (∼10-fold: supplementary fig. S2, Supplementary Material online) relative to the 2012-Assembly, with widths converging briefly between 200 and 100 ybp (66–33 gbp) before again exhibiting a reduction in the CI of the 2019-Assembly.

We observe minimal among-sample variation in demographic history as shown by the largely overlapping histories inferred by PSMC and MSMC (fig. 2). Interestingly, the only exception to this is that PSMC identified a single sample as having a distinct demographic history in which the decline first beginning during the penultimate deglaciation (fig. 2) is not followed by a recovery. However, this trajectory coalesces with that of the other samples ∼10 kybp.

#### Impact of Contiguity on Demographic Inference: Simulated Data

**Fig. 3.**
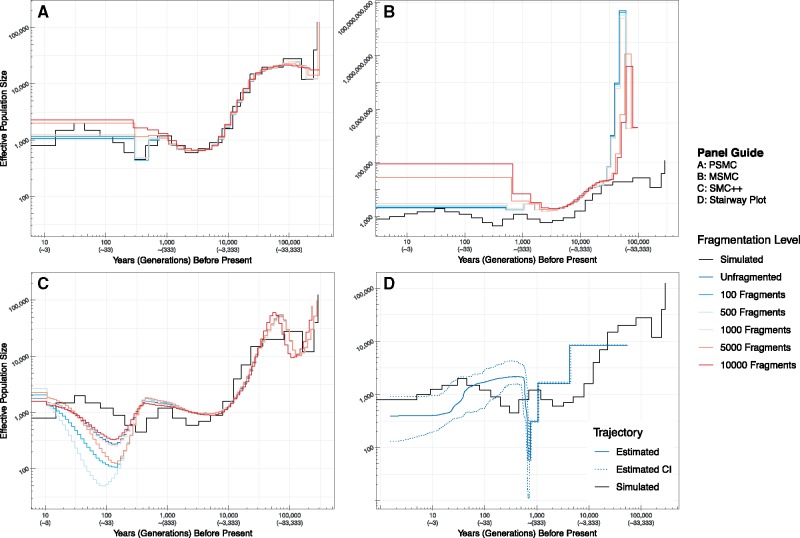
Estimates of the effective population size, *N*_e_, through time for simulated genomes fragmented to varying extents using PSMC, MSMC, SMC++, and Stairway Plot. Twelve whole genomes were simulated using MaCS in the form of six equal length (2.88 Mb) chromosomes (the same number of autosomal chromosomes in devils) assuming a demography intermediate to those inferred for Tasmanian devils (shown in fig. 2). The total length of the simulated sequence data is approximately equal to 90% of the length of the devil genome, the amount of sequence used in the empirical component of our study. The black stepped line in each panel is the simulated demography. Estimates obtained using less-fragmented genomes are shown in cooler colors, whereas more fragmented genomes are shown in warmer colors. Unfragmented trajectories are those obtained using the six simulated chromosomes as input. As each artificial fragment was of equal length, the true number of fragments in each set was 96, 498, 996, 4,998, and 9,996. Therefore, scaffold lengths in each fragmented set were approximately 2.91 Mb, 562 kb, 281 kb, 56 kb, and 28 kb, respectively. The Stairway Plot is only applied to the unfragmented genome as the site frequency spectrum of simulated data is insensitive to fragmentation (number of sites remains the same). Both years and number of generations (in parentheses) are reported on the *X* axis.

**Fig. 4.**
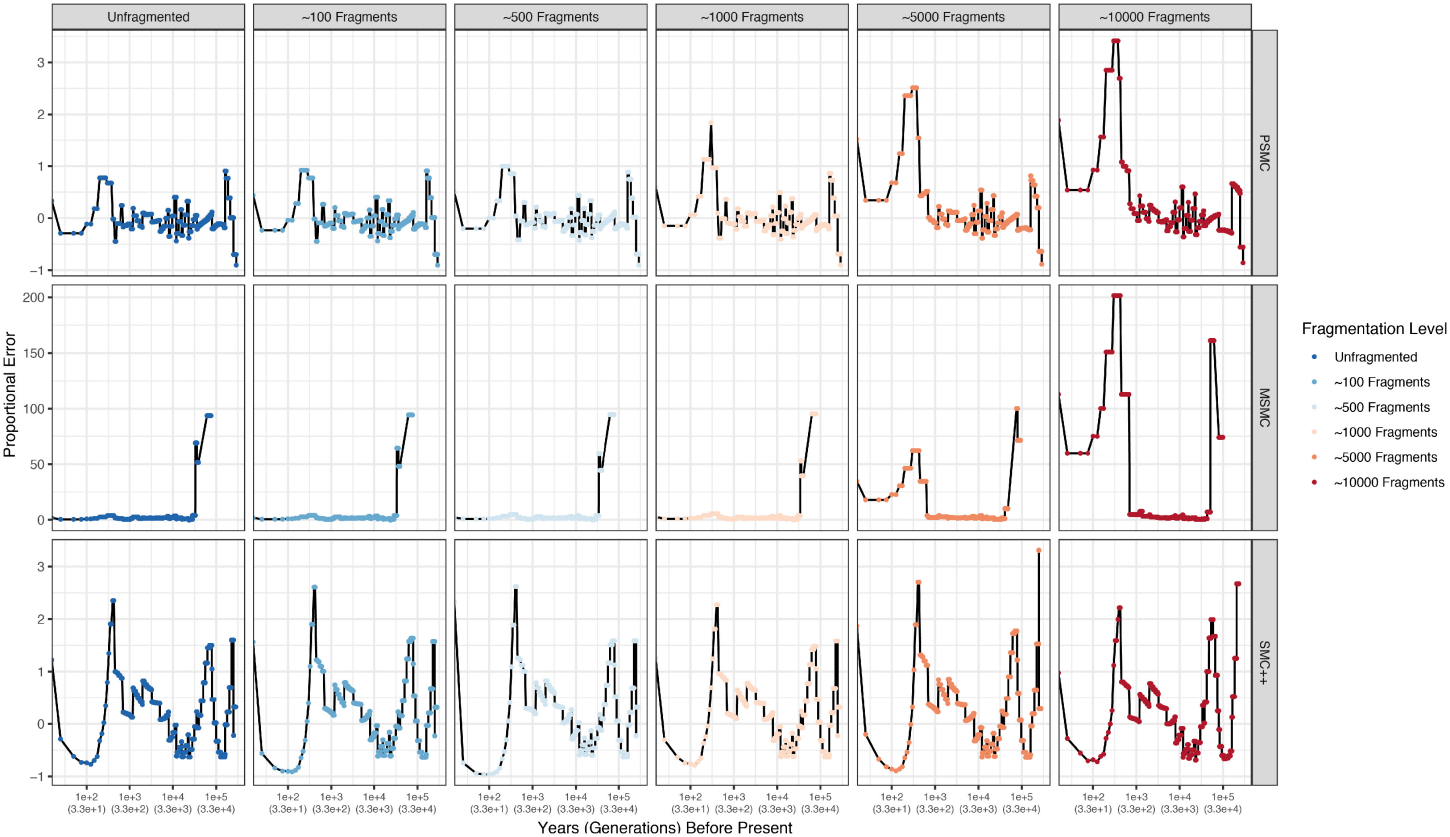
Proportional error for estimates of *N*_e_ through time for simulated genomes fragmented to varying extents using PSMC, MSMC, and SMC++. Rows depict results for different methods, whereas columns depict results for different fragmentation levels. Results for MSMC are truncated such that proportional errors >300 (observed exclusively between 10 and 33 kgbp) are not shown. Results were truncated to enable visualization of increased error in recent generations for more fragmented data sets. The upward trend toward the present in the first four columns for MSMC are due to significant overestimates that, although apparent in figure 3*B*, are washed out be the enormous overestimations beginning ∼10 kgbp.

Application of PSMC, MSMC, and SMC++ to simulated genomes varying in their degree of contiguity revealed that each method is broadly robust to genome assembly fragmentation (figs. 3 and 4). Population size inferences by PSMC toward the present (∼333–166 generations ago) become upwardly biased as the degree of fragmentation increases (figs. 3*A* and 4). The upward bias of recent estimates is most notable beginning at ∼5,000 fragments and becomes worse at ∼10,000 fragments. Similar behavior is exhibited by MSMC, with recent population size estimates spuriously increasing with increasing fragmentation. However, MSMC exhibits a pathological estimation of ancient (∼33–10 thousand generations ago), extreme population size fluctuations (figs. 3*B* and 4). In contrast with recent population size estimates, these spuriously inferred ancient population size fluctuations decrease in magnitude with increasing fragmentation. As with PSMC, this behavior becomes most significant beginning at ∼5,000 fragments and worsens as degree of fragmentation increases. SMC++ exhibits the least systematic relationship between degree of fragmentation and proportional error (figs. 3*C* and 4). However, SMC++ estimates a population decline ∼133 generations ago followed by recovery ∼42 generations ago. The magnitude of this decline is not related to the degree of fragmentation however. More importantly, this estimated decline does not correspond to the true population history. Broadly speaking, results obtained using the simulated and empirical data sets exhibited a high degree of similarity.

#### Comparison among Methods

Taken together, methods dependent on the Sequentially Markovian Coalescent (PSMC, MSMC, SMC++) produced demographic reconstructions that were qualitatively similar to one another (fig. 2). Note that SMC++ is a hybrid method, leveraging both the SMC and the SFS. Incongruence among these methods was, however, more dramatic toward their most recent and ancient timescales using both simulated and empirical data sets.

Notably, PSMC exhibits the greatest accuracy and precision of all methods (figs. 3*A* and 4). Unlike PSMC, accuracy of MSMC is low during intermediate (∼10,000–666 generations) time periods despite high precision (figs. 3*B* and 4), with population size being consistently overestimated. In fact, MSMC overestimates population sizes at every time step, regardless of the degree of fragmentation (figs. 3*B* and 4). As stated earlier, MSMC pathologically estimates extreme population size fluctuations between ∼33 and 10 thousand generations ago, with population sizes reaching between 400 million and ∼40 billion. This pattern is observed when using both empirical and simulated data (figs. 3 and 4). SMC++ exhibits accuracy worse than PSMC, but better than MSMC (figs. 3*C* and 4). Nonetheless, the precision of estimates is high until ∼133 generations ago, at which point the precision decreases in a manner unrelated to fragmentation.

The only method wholly reliant on the SFS, the Stairway Plot inferred a demographic history largely incongruent with those produced by SMC-based methods (figs. 3*D* and 4). Using empirical data, the initial decline takes place around the same time as inferred by other methods. The recovery that immediately follows is not inferred by other methods however. In contrast, the timing of the decline according to results using simulated data lags behind the actual population decline, whereas the recovery takes place too rapidly. Nonetheless, Stairway Plot is the only method to infer the shape of the documented recent Tasmanian devil population decline, suggesting potential utility for contemporary analyses.

## Discussion

### Genome Assembly Quality Does Not Impact Inference of Population Size History

Surprisingly, we found each method to be broadly robust to genome assembly quality (figs. 2 and 3). Importantly, this is true for methods based on the SMC, which based on prior expectations could be sensitive to variation in scaffold length distributions. Further, bootstrap estimation variance did not increase with increasing genome fragmentation for any method as assessed using empirical data (fig. 2).

Nonparametric bootstrapping is used by all methods discussed herein to assess parameter estimate uncertainty. This assumes that the method is estimating without error, given a sample (in this case a collection of sequences or SFS). We argue that future efforts would benefit from quantifying and reporting this estimation uncertainty. One possible remedy could be Bayesian sampling of a posterior distribution of local TMRCAs, leading to a subsequent posterior distribution of estimates of *N*_e_ at each time step. Alternatively, using methods derived from the SMC, the distribution of estimated local TMRCAs and associated population sizes could be instead represented as an empirical distribution of *N*_e_ through time for a given sample. We also are not aware of any method that implements an approach to test model adequacy. Parametric bootstrapping, through simulation under the fitted model and resampling of estimates could prove useful to accomplish this.

One methodological consideration that could impact our empirical results is the manner in which each method handles gaps. Because Dovetail Genomics inserts a gap of 100 *N*’s for every join it makes, our improved assembly (2019-Assembly) has many more gaps than does the 2012-Assembly (table 1), with each gap corresponding to a join between two of the 2012-Assembly’s contigs. Without *a priori* specification of gaps, this could lead SMC methods to interpret that contigs comprising the input scaffolds are closer than they are. Further, these gaps may be interpreted as runs of homozygosity, leading to the estimated population sizes being artificially depressed. Interestingly, we see little evidence that the greater number of gaps in the 2019-Assembly had an impact on our results, as inferences obtained using each assembly are extremely similar. Further, inferences are qualitatively similar between those obtained using simulated and empirical data. This instills further confidence that our results are robust to the handling of gaps, as simulated data were generated assuming a demography approximately equal to that inferred using empirical data.

Application of each method to simulated genomes that were progressively fragmented to increasing degrees only further supported these findings. Accuracy of parameter estimates by methods leveraging the SMC worsened for recent (∼333 gbp) and more ancient (>30 kgbp) time periods beginning at ∼5,000 fragments. Most methods reliant on the SMC exhibit a high degree of precision between 50 kgbp and 333 gbp (but see MSMC), whereas precision subsequently decreased for more recent estimates.

### Use of SMC- or SFS-Based Methods Recovers Alternative Population-Size Histories

Whereas inferences were similar among genomes within each method, incongruent results were observed when comparing methods using either the SMC or SFS using both empirical and simulated data. Specifically, inferences made by the Stairway Plot were largely discordant with those made by PSMC, MSMC, and SMC++. In contrast, SMC++, which leverages both the SMC and the SFS, recovered results intermediate to the other SMC methods and the Stairway Plot toward the present, likely due to the conflicting signals picked up by these two approaches. These results were consistent with previous work showing methods dependent on the SMC suffer from difficulties in estimating recent population history, but excel in the distant past (Li and Durbin 2011; Liu and Fu 2015; Terhorst et al. 2017). In contrast, methods exclusively dependent on the SFS excel at inferring more recent history, but perform less well when estimating more ancient population size history (Liu and Fu 2015; Terhorst and Song 2015; Lapierre et al. 2017; Baharian and Gravel 2018; Rosen et al. 2018).

An important caveat regarding the use of the SFS is its sensitivity to sample size. Specifically, numerous studies have demonstrated that the accuracy and precision of parameter estimates obtained using the SFS positively correlates with sample size (Adams and Hudson 2004; Keinan and Clark 2012; Robinson et al. 2014; Liu and Fu 2015; Terhorst and Song 2015). Likewise, it has been shown that for any sample size, parameter estimates are most error-prone for ancient demographic events. Importantly, this tendency is exacerbated by small sample size. However, increasing sample size has been shown to not only increase parameter estimate accuracy and precision for ancient demographic events but also for recent ones (Robinson et al. 2014; Liu and Fu 2015). In this context of our study, the discordance between inferences obtained using the Stairway Plot, which exclusively relies on the SFS, and the other methods may be explained by our low sample size. However, limited sample sizes for nonmodel organism whole-genome resequencing projects may be common (table 2).

### Geologic, Climatic, and Anthropogenic Drivers of the Demographic History of the Tasmanian Devil

We found evidence broadly supporting several hypothesized biogeographic, anthropogenic, and climatic drivers of the Tasmanian devil’s demographic history. First among these was a population decline during the penultimate deglaciation in which Tasmania was separated from mainland Australia (135–43 kybp Lambeck and Chappell 2001). Only SMC++ and PSMC detected this decline, with a larger reduction in *N*_e_ by ∼50,000 (fig. 2) detected by SMC++. Given the persistence of devils on the mainland following the re-emergence of the Bass Strait land bridge ∼43 kybp following the penultimate deglaciation, admixture among previously isolated mainland and island lineages may have masked some of the impacts of this event on genetic diversity (Chikhi et al. 2010).

Although ethnographic accounts indicate that Aboriginals may not have eaten carnivorous marsupials such as the Tasmanian devil (Owen and Pemberton 2005), it is possible that their presence may have negatively influenced devil population density and size via persecution and competition. Consequently, we hypothesize that the penultimate deglaciation and the colonization by Aboriginals each may have independently contributed to the observed population decline, or that it may have been their shared influence that initiated and maintained this decline. Although this hypothesis is compatible with our results, we cannot at this time tease apart the relative contributions of the anthropogenic and climatic/geologic factors.

During the LGM, sea surface temperatures were ∼4 °C cooler than at present, and climate in Tasmania was cooler and drier than prior to the LGM, with landcover being dominated by grassland and open shrub (Barrows and Juggins 2005). These climatic changes potentially restricted distributions of prey of the Tasmanian devil (Colhoun and Shimeld 2012; Brüniche-Olsen et al. 2014), and consequent submersion of the Bass Strait ∼14 kybp (Lambeck and Chappell 2001) likely restricted the distribution of devils. Indeed, we find a steepening of the decline of *N*_e_ across all methods consistent with these events. Following this most recent isolation of Tasmania from the continent and extinction of devils on mainland Australia ∼3.5 kybp (White et al. 2018), alleviation of bottlenecks in island devils by emigration from mainland populations was no longer possible. Furthermore, the TMRCA of Tasmanian and southern mainland populations of devils has been estimated at ∼26,400 ya using whole mtDNA genomes (Brüniche-Olsen et al. 2018). Notably, this timing is coincident with the LGM and corresponds to the large-scale decline in *N*_e_ estimated by most methods (fig. 2). Jointly, these factors lend additional support to the interpretation that the separation of Tasmania from mainland Australia during this time led to population subdivision and subsequent population decline. The severity of these declines is further explained by the documented coincident change in climate, and landcover. An alternative explanation for these declines is that for more ancient time periods (i.e., prior to the LGM), the population history being estimated could be for that of mainland populations. Following the LGM, inferred population size histories may be for Tasmanian population.

Extreme ENSO events 5–3 kybp led to periods of extended drought (Donders et al. 2008), further affecting devil population sizes via prey population declines (Brüniche-Olsen et al. 2014, 2018). Although the onset of ENSO events, increased populations of Aboriginals and the introduction of dingos have been implicated in the mainland extinction of devils, dingos were never introduced on Tasmania, and Tasmanian Aboriginal populations remained at lower densities than on the mainland (Brüniche-Olsen et al. 2018). Thus, only the impacts of the ENSO events are shared between Tasmanian and mainland populations of *S. harrisii* (Brüniche-Olsen et al. 2018), mirroring the drivers of mainland extinction of the thylacine (White et al. 2018). However, our results show that the ENSO events occurred near the end of the population decline (fig. 2*B*), suggesting that they are unlikely to have been the sole driver of the inferred decline.

As recently as 1830, European colonists made concerted efforts to reduce, if not exterminate, devils from rural areas, considering them as pests that threatened livestock (Owen and Pemberton 2005). Continued efforts to cull populations of devils resulted in the further reduction of population sizes. Not surprisingly, only the Stairway Plot detected the reduction in *N*_e_ around this time (fig. 2) due to the increased sensitivity of the SFS to recent demographic history. Methods dependent on the SMC detected to some degree an increase in *N*_e_ toward the present, with PSMC and MSMC unrealistically inferring present day population sizes in the millions (fig. 2). We believe these results to be an artifact of the SMC’s well-documented difficulty in estimating *N*_e_ toward the present (Sheehan et al. 2013; Liu and Fu 2015; Nadachowska-Brzyska et al. 2016). The limited increase inferred by SMC++ is likely due to an interaction between the SMC and SFS.

### Recommendations for Practical Application and Interpretation

Given that the SFS tends to perform better when inferring recent population history, and the SMC performs better when estimating more ancient history, we propose the following recommendation. Based on the results of the simulation portion of our study, PSMC is most reliable (i.e., least sensitive to fragmentation) between ∼100 kgbp and ∼333 gbp. In contrast, MSMC will be most reliable between ∼100 kgbp and ∼1,000 gbp, however, accuracy is low, with parameter estimates consistently overestimated. Based on our results, we recommend that results of MSMC for times older than 10 kgbp should not be interpreted. SMC++ will be most reliable between ∼100 kgbp and ∼166 gbp. However, PSMC is still more accurate overall across this time interval than SMC++. Broadly speaking results of SMC methods for times more recent than 333–166 gbp should be interpreted with caution. For more ancient inferences, we recommend cautious interpretation of estimations later than 10 kgbp for MSMC, 17 kgbp for SMC++, and 33 kgbp for PSMC. Although present-day parameter estimates are close to their true values (albeit reliably overestimated), the population size trends are either uninformative or misleading. The Stairway Plot is the only method tested that accurately reconstructs the shape of very recent (∼16 gbp–present) population size fluctuations. However, due to inconsistency between the results of Stairway Plot analyses conducted using simulated and empirical data, we recommend careful interpretation of these results.

### Summary of Devil Demographic History

The Tasmanian devil has experienced staged population declines since the penultimate deglaciation, ∼143 kybp, likely due to a combination of: repeated isolation and reconnection to the Australian mainland, changing climate that caused periods of cooling and later drought that restricted Tasmanian devil and prey distributions, competition and potential persecution by Aboriginal Australians, with recent culling by European colonists. Although inferences made by the Stairway Plot more closely reflect recent population size trends, parameter estimation should be interpreted with caution. Specifically, the results of our simulations indicate that the Stairway Plot tends to underestimate contemporary population sizes, despite most-accurately estimating the shape of recent trends. Regardless, it appears that the long history of decline in devils may have led to increased susceptibility to transmissible cancers (DFTD, DFT2) due to a loss of standing genetic diversity.

Importantly, sampling design may impact demographic reconstruction. Previous work has explored the factors that may confound the estimation of *N*_e_ by genomic methods such as those that rely on the SMC and SFS. For instance, population structure and gene flow among demes has been repeatedly shown to bias demographic inference by generating spurious patterns of population expansion (Nielsen and Beaumont 2009; Chikhi et al. 2010; Paz-Vinas et al. 2013; Mazet et al. 2015, 2016). Specifically, sampling two demes with ongoing gene flow tends to generate a pattern of population expansion (Mazet et al. 2016). In contrast, sampling only one of these demes leads to the erroneous inference of a population bottleneck (Nielsen and Beaumont 2009; Mazet et al. 2016). In response, some methods have attempted to account for this by estimating independent demographic histories and timing of population splits for samples obtained from different present-day populations (e.g., MSMC, SMC++). However, we know of no method/approach that accounts for the reverse of this situation, in which ancient populations merged into a single contemporary population.

Our sampling of devils is not spatially representative of the whole of the species’ range. Our 12 samples are limited to the North-West-central portion of Tasmania, whereas devils are broadly distributed across the extent of the island, excepting the South-eastern third. Importantly, our sampling occurs within a single population according to previous population genetic studies (Hendricks et al. 2017). Consequently, there is little concern that we have sampled individuals from two contemporary demes. The potential impacts of this sampling design on our results are 2-fold. First, this limits the confounding effects of conflicting demography on our inferences. Second, it limits our ability to detect the demographic consequences of DFTD, which was first detected in the north-eastern corner of Tasmania ∼22 years (∼7 generations) ago (Hawkins et al. 2006). As the disease only arrived in the region from which our samples were taken ∼8 years (<3 generations) ago, we are unable to detect the consequences of this disease on genetic diversity in our samples, despite well described population declines (Lachish et al. 2007; Hamede et al. 2015). However, this fact also means that our results are unlikely to be confounded by recent changes driven by DFTD.

With the exception of the one sample inferred by PSMC to have a slightly different history (fig. 2*B*), estimates for each sample (faint lines) largely overlap. As is, we have no hypothesis as to why this individual, one of the seven samples obtained from Takone (the most north-westerly population sampled) might have a different demographic history. The close geographic proximity of the remaining 11 samples likely explains the minimal variation in demographic history among them however.

Note also that our estimates represent a lower bound for timing of the population size history, because they are derived from a genome-wide estimate of the substitution rate between the Tasmanian devil and three other closely related marsupials (*Macrophus eugenii*, *Phascolarctos cinereus*, *Monodelphis domestica*). Consequently, the true value of the mutation rate may be even greater, pushing the timing of population size shifts toward the present. In fact, such a shift would result in even greater concordance between biogeographic/anthropogenic events and observed population size history.

Importantly, we observe a number of consistencies between results obtained using empirical and simulated data. First, MSMC infers a spurious, enormous population size fluctuation between ∼100–30 kybp. MSMC also consistently estimates larger population sizes than other methods. We can confidently say that this is not due to the approach used for genotyping (samtools), as we also used these data for analysis with PSMC, yet PSMC performed much more consistently and in a manner consistent with results obtained using simulated data. Were the genotyping approach to be the cause of MSMC’s poor performance, we would expect similarly poor performance from PSMC, but we do not observe this. Second, SMC++ appears to consistently (using both empirical and simulated data) estimate a population size decrease and recovery between 500 ybp and the present day. In the case of estimates obtained using simulated data, this decline is inconsistent with the history used to simulate the input sequences. Thus, we do not interpret literally these recent declines inferred by SMC++ using empirical data. Lastly, the Stairway Plot infers a demography incompatible with those inferred using other methods. Again, this holds for both inferences obtained using empirical and simulated data.

## Conclusion

In this study, we used four methods designed to infer population size history from genomic data sets and assessed their performance using genome assemblies varying in their degree of contiguity. We accomplished this using both empirical genome assemblies, as well as simulated genomes that were artificially fragmented. We found minimal impacts of genome assembly on inferred patterns of historical *N*_e_, suggesting that conservation genomic studies leveraging fragmented assemblies will be capable of producing robust inferences. Depending on the goals of a study, multiple classes of methods should be used. If only recent (<16 gbp) demographic history is to be assessed, methods based on the SFS should be used. Additionally, traditional population genetic studies will continue to be invaluable in these contexts. However, if more ancient histories (>16 gbp) are the focus of study, SMC methods are preferable.

## Materials and Methods

### Genome Assembly

We extracted ∼4 μg of high-quality genomic DNA from a captive, adult female Tasmanian devil from the San Diego Zoo. Note that, this was a different individual than was sequenced for the 2012-Assembly, however, the individuals came from the same geographic regions (North-eastern Tasmania) and a single interbreeding population (Hendricks et al. 2017). Consequently, we anticipated minimal differences outside of slight variation in the number/distribution of variable sites when aligning resequenced genomes to the two assemblies. Two sequencing libraries were constructed using the Chicago library protocol by Dovetail Genomics (Santa Cruz, CA) and run on a single lane of paired-end 150 bp sequencing on an Illumina HiSeq X machine. Scaffolding of the assembly was conducted with the Murchison et al. (2012) genome as an input assembly in the Dovetail HiRise assembly pipeline.

### Sampling and Whole-Genome Sequencing

Individuals were nonlethally trapped as previously described (Hamede et al. 2015; Margres, Jones, et al. 2018). We sequenced the genomes of 12 *S. harrisii* from northwestern Tasmania (fig. 1), 10 of which are included in Margres, Ruiz-Aravena, et al. (2018). Genomic DNA was extracted from ear biopsies. Whole-genomes for each individual were sequenced with 150 bp paired-end on an Illumina HiSeq X platform to ∼30× coverage; sequencing was performed at the Northwest Genomics Center at the University of Washington (Seattle, Washington) and GENEWIZ (South Plainfield, NJ).

### Alignments and Variant Calling

To obtain SNPs across all samples, data were processed as previously described Margres, Ruiz-Aravena, et al. (2018). Briefly, raw reads were merged with FLASH2 (Magnoc and Salzberg 2011) and trimmed with Sickle (Joshi and Fass 2011). Reads were aligned to the previously published reference genome (Murchison et al. 2012) (downloaded from Ensembl January 2016) as well as the new genome assembly described in this study using the BWA-MEM algorithm (Li and Durbin 2009). Duplicate reads were marked and removed using Picard (Broad Institute 2018). Joint genotyping of all 12 samples was performed in GATK according to their best practices (McKenna et al. 2010; DePristo et al. 2011) using HaplotypeCaller for each reference genome independently as previously described (Margres et al. 2017). Briefly, only variants matching any of the following criteria were retained: quality by depth (QD) ≥ 2.0, Phred-scaled *P* value (FS) ≤ 60.0 and root mean square of the mapping quality (MQ) ≥ 40.0. Variants with mapping quality rank sum test approximation (MQRankSum) of 12.5, and a read position rank sum test approximation (ReadPosRankSum) of 8 were removed. We identified 2,822,764 raw SNPs and 1,194,776 raw indels when aligning to the previously published reference genome (Murchison et al. 2012). We identified 2,790,031 raw SNPs and 1,189,816 raw indels when aligning to the new genome assembly described in this study.

These raw SNPs were subsequently filtered more stringently using vcftools v0.1.14 (Danecek et al. 2011), retaining only biallelic sites matching the following criteria: genotype quality ≥ 60 (minGQ 60), mean depth of coverage ≥ 15 (min-meanDP 15), present in at least 75% of samples (max-missing 0.75). Filtering in this way reduced the number of SNPs recovered to 1,468,034, and 1,468,044 in the 2012- and 2019-Assemblies, respectively.

To obtain sample-specific SNP data sets for use with PSMC and MSMC, we used the samtools mpileup and bcftools call functions, removing sites with mean map and base qualities <20 (samtools mpileup -q 20 -Q 20 -C 50 -u). Samtools differs from GATK in that uses individual samples for genotype calls, and thus does not incorporate population allele frequencies into its genotype calls. Likewise, it makes no assumptions regarding Hardy–Weinberg equilibrium. We chose this approach, as both MSMC and PSMC are applied in our study to individual samples, and so we sought to maximize the number of unique sites per sample. Further, this samtools workflow has become standard for studies using PSMC or MSMC (see Nadachowska-Brzyska et al. 2016).

To test for differences between SNP calling procedures, we used the bcftools isec command(Li et al. 2009) to identify the number of shared and unique sites among approaches for each sample. These results are summarized in supplementary table S3, Supplementary Material online. To further test whether our results are robust to our genotype calling approaches, we applied PSMC to data sets for one sample (Sample 1, table 2) obtained using both GATK and Samtools (supplementary fig. S4, Supplementary Material online). Results were qualitatively similar and thus not discussed further. Note that results for samtools plotted in supplementary figure S4, Supplementary Material online, are the same as in figure 2.

### Calculation of the Mutation Rate

To obtain an estimate of the per-generation mutation rate, a parameter needed a priori by all demographic reconstruction methods used in this study, we followed a two-stage procedure. First, genome-wide distributed estimates of the synonymous substitution rate (d*S*) were obtained from the 2012-Assembly of *S. harrisii*, and the orthologous coding sequences of the closely related marsupials for which whole genome data are available (*Macrophus eugenii*, *Phascolarctos cinereus*, *Monodelphis domestica*) using the method of Nei and Gojobori (1986). From this distribution of d*S* values, we obtained a point estimate as the median of this distribution. This rate (per-site) was rescaled to units of time by pruning the time-calibrated phylogenetic tree of Mitchell et al. (2014) to these four taxa to obtain the branch-length (in number of years) from *S. harrisii* to the other three taxa. This was subsequently rescaled to generations by dividing this value by three, the generation time of *S. harrisii* (Pemberton 1990). By dividing our median estimate of d*S* by this branch length (in units of generations), we obtained an estimate of the per generation substitution rate (1.394808e-08). We use this as a conservative lower bound estimation of the mutation rate.

### Inference of the SFS

The Stairway Plot method requires as input a SFS. To infer a folded SFS (in which only minor alleles are considered) we used ANGSD (Nielsen et al. 2012). ANGSD implements a two-stage procedure to infer the SFS. Briefly, ANGSD first calculates individual genotype likelihoods from the.bam assemblies assuming Hardy–Weinberg Equilibrium (HWE: option -doSaf 1) and uses these to calculate the sample allele frequencies at each site. Using these genotype likelihoods, a maximum likelihood estimate of the SFS is obtained through subsequent iterative application of an Expectation Maximization (EM) algorithm (program realSFS; Nielsen et al. 2012). We filtered such that bases with meaningless map quality and poor Qscores were removed (-minMapQ 1 -minQ 20). We repeated this procedure using the resequenced genomes aligned to both the 2012- and the 2019-Assemblies.

### PSMC Analysis

PSMC was the first method developed that uses the SMC to infer historical population sizes. A single input file is used as input for a given analysis. Consensus fastq files were produced using the vcf2fq command implemented by the vcfutils.pl script distributed by bcftools-1.3.1 using the snps obtained by samtools before being converted to PSMC input files using the fq2psmcfa command. Estimates were conducted using the single input file per sample. All estimates were run using 50 EM iterations.

To conduct bootstrap estimates, we first fragmented long scaffolds using the splitfa function distributed with PSMC. The splitfa function fragments long scaffolds into 5 Mb long segments. Estimates were then conducted using the psmc -b option. Ten bootstrap estimates were made per sample, resulting in a total of 120 bootstrap replicates.

### MSMC Analysis

Although MSMC allows for demographic inference using multiple individuals, it is computationally limited in the number of haplotypes it can take as input (maximum of eight, or four diploid individuals). Due to the large number of possible combinations of individuals and resultant immense number of necessary bootstrap replicates for our sample size, we chose to produce estimates using each of our 12 individuals. Although MSMC requires phased data when applied to more than one sample, it does not when applied to a single sample. As such, we did not attempt to phase our data for this analysis. MSMC requires individual input files per chromosome, or in our case, scaffold. Thus, we produced an MSMC input file per individual, per scaffold with a length greater than that of the N90. Input files were generated by converting the SNPs obtained using samtools to MSMC input format using the bamCaller.py script. Estimates were conducted using the full set of input files.

To conduct bootstrap estimation, scaffolds >5 Mb were broken up into a number of smaller segments (as done by default in PSMC) such that the resultant fragments were equal in size. This resultant set of fragmented scaffolds was then sampled with replacement until a resampled genome was produced of equivalent length. As our MSMC estimates were for each individual, we conducted 10 bootstrap estimates for each individual, producing a total of 120 bootstrap replicates. All estimates were conducted using 50 EM iterations.

### SMC++ Analysis

Unlike the Stairway Plot, SMC++ leverages structural/linkage-based information in its estimates of historical demography, however, it does still incorporate the SFS in its estimate. As we lack positional data relative to chromosomes for the 2019-Assembly, we treated each assembly as a series of scaffolds. Thus, although unable to investigate demographic history at a chromosome by chromosome basis, we can be sure that any observed runs of homozygosity are real and not a consequence of missing data among scaffolds.

SMC++ leverages both the SFS as well as the SMC and requires a single input file per scaffold. Rather than calculating the SFS relative to the reference genome, SMC++ uses a single “distinguished individual” selected from the pool of samples against which all other samples are compared. As the choice of distinguished individual under this model may impact demographic inference, we generated 12 SMC++ files per scaffold, each differing in which individual is distinguished, leaving the other 11 as “undistinguished.” Consequently, use of all scaffolds would result in the need to specify as input ∼420,000 input files in the case of the 2012-Assembly. Attempts to do so resulted in the program crashing. Therefore, we chose instead to restrict our analysis to the N90 set of scaffolds. Doing so reduced the 2019-Assembly to 422 scaffolds, whereas the 2012-Assembly was only reduced to 3,263 scaffolds. To ensure consistency, we used the N90 set of scaffolds for all analyses.

We converted each scaffold into an SMC++ formatted input file using the vcf2smc script distributed with SMC++. All SMC++ input files were used together in a single run, resulting in a composite likelihood estimate, integrating across the variability generated by choice of distinguished individual. Runs were conducted assuming the previously described mutation rate, 40 knots (potential inflection points), thinning every 2,000 sites, and 50 EM iterations. Estimates were restricted to be made between 10 and 750,000 generations since present.

To bootstrap, we used the same sets of fragmented and resampled scaffolds as in our MSMC analysis. Bootstrap estimates were conducted using 25 such sets produced in this way. This number was chosen due to the extreme computational resources demanded by SMC++ (estimates for the 2019-Assembly took nearly 50 days to complete on a 28 core machine, and nearly double that for the 2012-Assembly).

### Stairway Plot Analysis

Using the SFS described above, we proceeded to infer historical population demography using the Stairway Plot. All settings described here were applied to analyses of the SFS obtained from the resequenced sample aligned to either the 2012- or 2019-Assembly. To obtain estimates of *N*_e_ through time, 1,000 random subsamples of two-thirds of all sites were obtained from the empirical SFS. Individual Stairway Plots were estimated from these subsamples using a random selection of break points ranging from 3 to 24 in intervals of 3. These break points may be thought of as points at which *N*_e_ changes in time. We report the median of these estimates as our final, as well as the upper (97.5%) and lower (2.5%) bounds as our pseudo-CI.

### Simulation, Fragmentation, and Analysis of Genome-Scale Sequences

To further test the robustness of the methods used herein to genome assembly contiguity, we replicated the previously described analyses on simulated genomes. We assumed a demography intermediate to those inferred from empirical data (fig. 3, black lines). To accomplish this, we used MaCS (Chen et al. 2009). Specifically, we simulated six chromosomes of equal size (480 Mb), such that the sum of their lengths was approximately equal to 90% of the devil genome (length of sequence used in the empirical component of this study). The scaled mutation rate (*θ*  =  4*Nμ*) was established using the mutation rate derived in this study, whereas the scaled recombination rate (*ρ*  =  4*Nr*) was based on the estimate of per-generation recombination rate by Feigin et al. (2018). Importantly, MaCS requires that both *μ* and *r* are scaled by the length of the sequence being simulated (in our case 480 Mb). Thus, for our simulations, *θ*  =  0.00697404, and *ρ*  =  0.004573798. We simulated 12 diploid genomes in this way to maximize comparability between our simulated and empirical results. Simulated sequences were converted to VCF format using custom scripts.

We aimed to use these simulated genomes to test the impact of genome fragmentation on demographic inference. As such, we fragmented the simulated genomes to six levels, each varying in their degree of contiguity. We produced data sets comprised of 100, 500, 1,000, 5,000, and 10,000 fragments of equal length using SnpSift split (Cingolani et al. 2012), which splits a VCF file into fragments of specified length. As we split each chromosome into an equal number of fragments, rounding became necessary, leading the true number of fragments in each data set to equal 96, 498, 996, 4,998, and 9,996 fragments. This procedure produces VCFs in which variant sites retain their original position, regardless of the length of sequence actually contained within the file. Consequently, we rescaled the positions of the sites within each VCF proportional to the length of the sequence contained in that file. Failure to do so results in the methods used herein to assume long runs of homozygosity preceding the first site.

For each data set (including the unfragmented, chromosome level sequences), we produced input files for PSMC, MSMC, and SMC++. Note that, we only applied the Stairway Plot to the unfragmented data set because fragmentation of simulated data will not impact the SFS. In contrast, increasing fragmentation of empirical genome assemblies may lead to lower rates of alignment and thus fewer SNPs identified postfiltering. PSMC input files were produced using the smc2psmc.py script distributed with SMC++. Specifically, a single-sample SMC++ input file was produced and subsequently converted to PSMC format. MSMC input files were produced using the generate_multihetsep.py script distributed with MSMC. SMC++ input files were produced using the vcf2smc command in SMC++. Lastly, the folded SFS was calculated directly from the multisample VCF of all 12 simulated, unfragmented diploid genomes using the vcftools –freq command.

We replicated the empirical analyses using each of the aforementioned data sets. Note that, we did not conduct bootstrapping of simulated data sets, due to both time and computational limitations. To determine the extent to which each method was sensitive to genome assembly contiguity, we calculated the proportional error $\left(=\frac{\mathrm{Estimated}-\mathrm{Simulated}}{\mathrm{Simulated}}\right)$ through time for each method and each fragmentation level (fig. 4). We chose to use this metric it retains the sign of error (i.e., communicates whether parameters were over- or underestimated), and because it may be intuitively interpreted in terms of percent error.

## Supplementary Material

Supplementary data are available at *Molecular Biology and Evolution* online.

## Supplementary Material

msz191_Supplementary_Data
