# Supplementary material for: Contemporary Demographic Reconstruction Methods Are Robust to Genome Assembly Quality: A Case Study in Tasmanian Devils
Source: Mol Biol Evol. 2019 Aug 19;36(12):2906–21. doi: 10.1093/molbev/msz191 (PMC6878949; doi:10.1093/molbev/msz191)
Supplement: msz191_Supplementary_Data [file msz191_supplementary_data.zip › Patton_et_al_2019_MBE_Supplement.pdf]

Contemporary demographic reconstruction methods are robust to genome assembly quality: A case study in Tasmanian Devils

Austin H. Patton,[*,1] Mark J. Margres,[1,2] Amanda R. Stahlke,[3] Sarah Hendricks,[3] Kevin Lewallen,[3] Rodrigo K. Hamede,[4] Manuel Ruiz-Aravena,[4] Oliver Ryder,[5] Hamish I. McCallum,[6] Menna E. Jones,[4] Paul A. Hohenlohe,[3] and Andrew Storfer[*,1]

[1]School of Biological Sciences, Washington State University, Pullman, Washington, USA

[2]Department of Organismic and Evolutionary Biology, Harvard University, Massachusetts, USA

[3]Institute for Bioinformatics and Evolutionary Studies, University of Idaho, Moscow, Idaho, USA

[4]School of Natural Sciences, University of Tasmania, Hobart, Australia

[5]Institute for Conservation Research, San Diego Zoo, San Diego, California

[6]School of the Environment, Griffith University, Nathan, Australia

***Corresponding author:** E-mails: austin.patton@wsu.edu, astorfer@wsu.edu

Associate Editor: Robb Brumfield

Article

© The Author 2013. Published by Oxford University Press on behalf of the Society for Molecular Biology and Evolution. All rights reserved. For permissions, please email: journals.permissions@oup.com

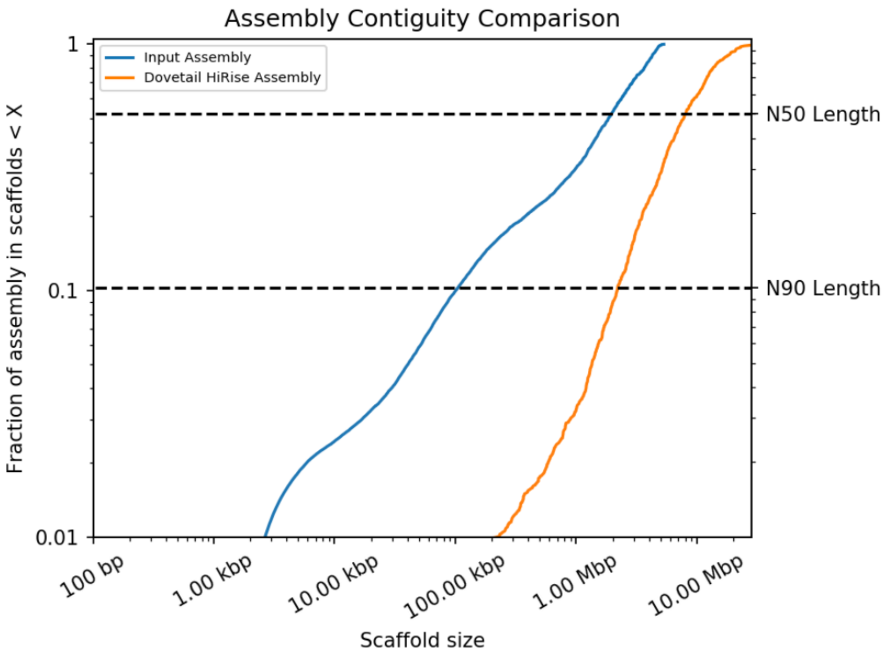

FIG. S1. A comparison of the contiguity of the input assembly and the final HiRise scaffolds. Each curve shows the fraction of the total length of the assembly present in scaffolds of a given length or smaller. The fraction of the assembly is indicated on the Y-axis and the scaffold length in basepairs is given on the X-axis. The two dashed lines mark the N50 and N90 lengths of each assembly. Scaffolds less than 1 kb are excluded. Figure courtesy of Dovetail Genomics.

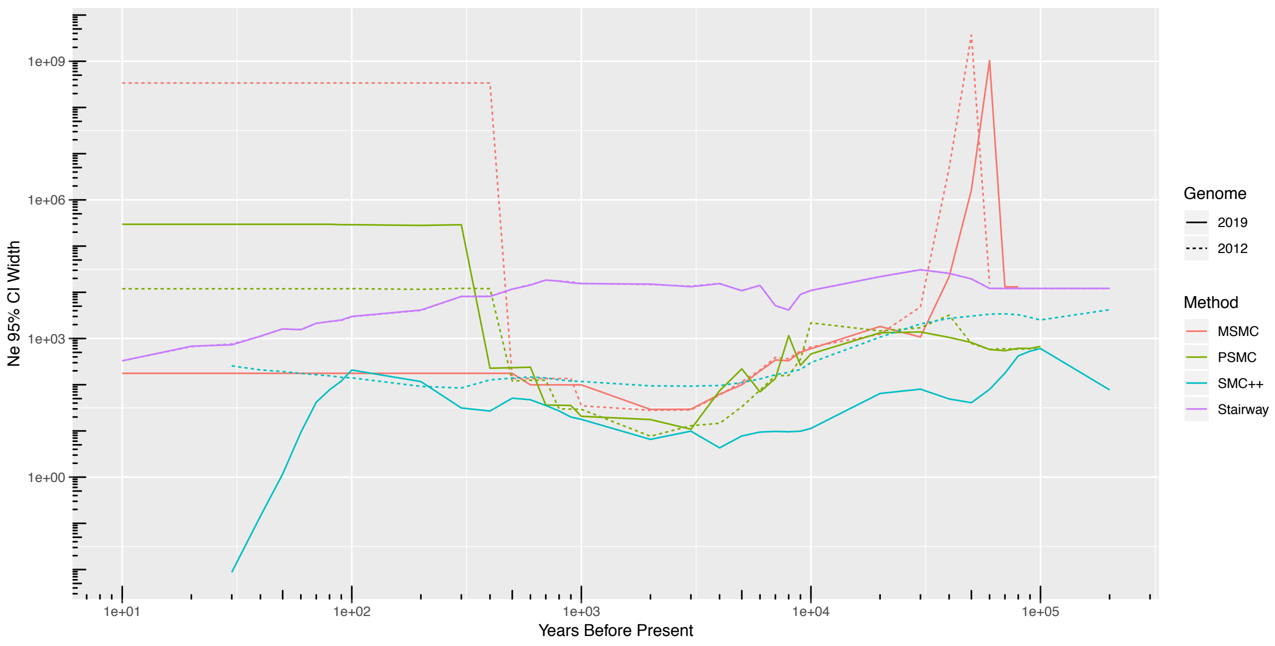

FIG. S2. Width of 95% confidence intervals in units of N_e from bootstrapped estimates for each method and from data aligned to each reference genome assembly. Colors correspond to method, whereas line type (dashed or solid) correspond to genome.

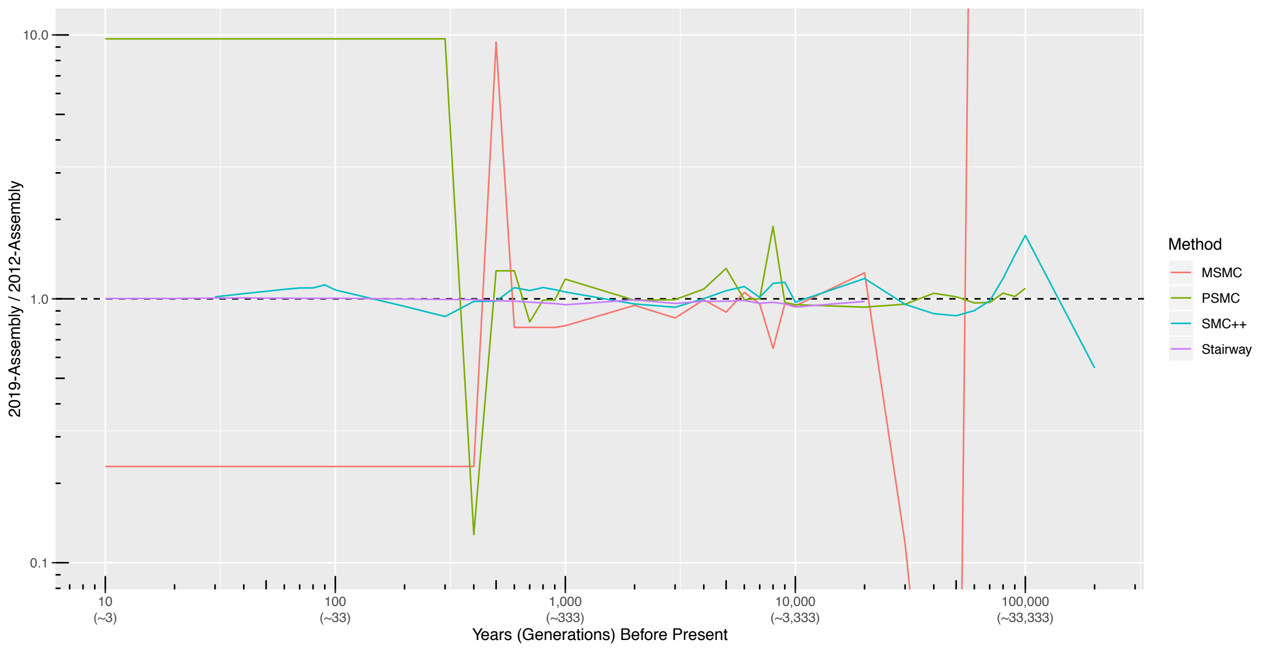

FIG. S3. Ratio between estimates of N_e obtained using the 2019-Assembly and 2012-Assembly. Positive values indicate that the 2019-Assembly produced larger estimates of N_e than the 2012-Assembly at a given time, whereas negative values imply the converse. Colors correspond to method, whereas line type (dashed or solid) correspond to genome. The Y-axis is truncated so as to assist visualization of the smaller differences between assemblies observed for methods other than MSMC.

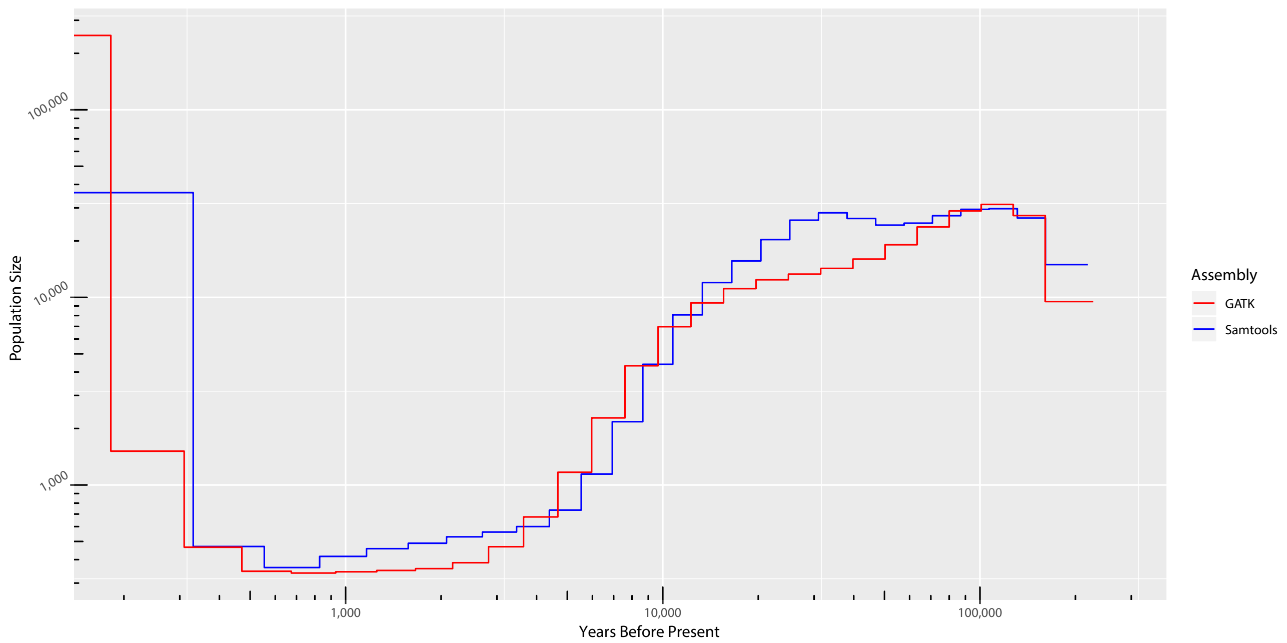

FIG. S4. Comparison of results obtained by PSMC using datasets generated using either the GATK or Samtools workflow using the 2019-Assembly. Results for a single sample are shown. Note that the history obtained by samtools is one of those plotted in Figure 2.

Table S1. Proportion of BUSCO (Benchmarking Universal Single-Copy Ortholog) genes found in each Tasmanian devil assembly using the mammalia odb9 dataset. Genes are split into four categories: complete and single-copy, complete and duplicated, fragmented, and missing.

Assembly	Percent Complete	Single Copy (%)	Duplicated (%)	Fragmented (%)	Missing (%)	Number of Genes Tested
2012-Assembly	89.5	88.8	0.7	5.7	4.8	4,104
2019-Assembly	91.0	90.2	0.8	4.6	4.4	4,104

Table S2. Summary of read-mapping and depth of coverage when aligning resequenced samples to the 2012- and 2019-Assemblies.

	2012-Assembly				2019-Assembly			
Sample	Total # Reads	Reads Mapped	Properly Paired	Mean Depth of Coverage	Total # Reads	Reads Mapped	Properly Paired	Mean Depth of Coverage
1	487816219	476249703 (97.63%)	297254080 (83.91%)	22.7703	487810393	476250154 (97.63%)	297428506 (83.96%)	24.7005
2	754390154	738157855 (97.85%)	601686738 (91.61%)	33.65	754413606	738216542 (97.85%)	602123110 (91.68%)	36.4703
3	772357502	755679072 (97.84%)	598792930 (90.46%)	34.551	772369449	755749615 (97.85%)	599282878 (90.53%)	37.4448
4	582285246	569757635 (97.85%)	457658986 (91.22%)	26.0071	582297330	569791198 (97.85%)	458066584 (91.30%)	28.1911
5	762110769	746012467 (97.89%)	610562718 (90.70%)	33.5666	762117924	746062967 (97.89%)	611319542 (90.81%)	36.3731
6	564302392	551760849 (97.78%)	457361944 (91.32%)	24.7102	564318840	551828927 (97.79%)	457668322 (91.38%)	26.7892
7	496983732	485996234 (97.79%)	323633856 (84.87%)	22.8975	496983287	486002143 (97.79%)	323786892 (84.91%)	24.8244
8	462236400	453012784 (98.00%)	298251344 (87.59%)	22.452	462235798	453018012 (98.01%)	298425690 (87.64%)	24.3388
9	467658531	458722897 (98.09%)	317214098 (87.16%)	22.3286	467659261	458728502 (98.09%)	317369320 (87.21%)	24.2071
10	496880012	486322051 (97.88%)	295175474 (86.63%)	24.1448	496879706	486325611 (97.88%)	295322956 (86.68%)	26.1805
11	488970774	479166892 (97.99%)	310804892 (86.69%)	23.5861	488972459	479172365 (98.00%)	310935310 (86.73%)	25.5728
12	505693050	495236752 (97.93%)	318022580 (86.19%)	24.5255	505679886	495229667 (97.93%)	318273728 (86.26%)	26.5831

Table S3. Summary of the shared and unique SNPs called by Samtools GATK. All numbers are counts of SNPs with the exception of the last two columns, which are percentages. Percentages represent the proportion of the grand total each genotyping method comprises.

Sample	Unique to Samtools	Unique to GATK	Shared	Samtools Total	GATK Total	Grand Total	% Samtools	% GATK
1	1075659	335840	631810	1707469	967650	2043309	52.64	47.36
2	1140780	343193	658324	1799104	1001517	2142297	53.25	46.75
3	1155429	343852	683999	1839428	1027851	2183280	52.92	47.08
4	1108209	341847	668659	1776868	1010506	2118715	52.31	47.69
5	1172778	349574	685945	1858723	1035519	2208297	53.11	46.89
6	1137928	333732	668470	1806398	1002202	2140130	53.17	46.83
7	1101919	337133	646455	1748374	983588	2085507	52.84	47.16
8	1140765	347415	612221	1752986	959636	2100401	54.31	45.69
9	1124921	343960	641058	1765979	985018	2109939	53.32	46.68
10	1070866	347692	653040	1723906	1000732	2071598	51.69	48.31
11	1099865	345649	672007	1771872	1017656	2117521	51.94	48.06
12	1118774	347534	665856	1784630	1013390	2132164	52.47	47.53
